# Implementing total skin electron irradiation in radiotherapy: a structured change management approach

**DOI:** 10.1007/s00066-025-02408-w

**Published:** 2025-05-14

**Authors:** Andrea Baehr, Maximilian Grohmann, Volker Platz, Nina Booken, Cordula Petersen

**Affiliations:** 1 Department of Radiation Oncology, University Medical Center Hamburg-Eppendorf, Hamburg, Germany; 2 Department of Dermatology, University Medical Center Hamburg-Eppendorf, Hamburg, Germany

**Keywords:** Mycosis fungoides, Cutaneous lymphoma, Total skin electron irradiation, Change management, Implementing new techniques

## Abstract

**Background:**

Total skin electron irradiation (TSEI) is a specialized radiotherapy technique used to treat cutaneous T‑cell lymphoma, including mycosis fungoides and Sézary syndrome. Despite its clinical benefits, TSEI is rarely implemented in clinical practice due to significant technical and organizational challenges. This study explores a structured approach to integrating TSEI into clinical routines by applying a comprehensive change management framework in a German radiotherapy department.

**Methods:**

The implementation process was based on Kotter’s eight-step change management model and carried out from 2022 to 2024 by a multidisciplinary team. Key steps included *creating a sense of urgency, forming a guiding coalition, developing and communicating a clear vision, and pilot testing to generate short-term successes*. A dynamic stakeholder analysis was conducted to continuously identify and manage factors that could promote or hinder the change.

**Results:**

The structured approach facilitated integration of TSEI into routine practice. The project enabled comprehensive staff training, adaptation of technical workflows, and development of necessary protocols and equipment. Key milestones were achieved, including initial patient treatments and positive staff feedback, demonstrating the method’s feasibility and acceptance. The stakeholder analysis was instrumental in reducing apprehensions and maintaining alignment among team members.

**Conclusion:**

This study demonstrates that a structured change management approach is effective in integrating complex techniques like TSEI into clinical practice. The findings highlight that a systematic framework can help to overcome organizational and technical challenges, thereby enhancing patient care and operational efficiency. Future efforts should continue to employ structured methodologies for the sustainable integration of new medical technologies.

**Supplementary Information:**

The online version of this article (10.1007/s00066-025-02408-w) contains supplementary material, which is available to authorized users.

## Introduction

Implementing new medical techniques within clinical settings is inherently challenging, requiring both technical proficiency and effective change management strategies. The implementation of complex techniques often faces significant hurdles, including resistance to change from staff, the need for extensive training, and the necessity of reconfiguring existing workflows. One advanced technique in radiation oncology (RO) is total skin electron irradiation (TSEI), primarily used to treat cutaneous T‑cell lymphoma. This technique offers remarkable positive results for patients with mycosis fungoides or Sézary syndrome [1, 2]. Despite its clinical benefits, the adoption of TSEI in many healthcare facilities remains limited due to the complexities involved in its implementation [3]. In the context of radiotherapy, there is a notable lack of detailed accounts and studies on the implementation of new techniques such as TSEI, particularly from a change management perspective. This gap in the literature is significant because the successful integration of TSEI into clinical practice involves not only technical adjustments but also organizational changes, training, and coordination among multidisciplinary teams.

While the value of structured change models is recognized, practical applications within the specific context of RO remain underexplored. References to change management models, such as those by Kotter, often appear as ancillary mentions in conclusions or as part of future project recommendations rather than as central themes [4, 5]. This paper aims to address this gap by providing a comprehensive account of how a structured change management approach was utilized to implement TSEI in a German radiotherapy clinic. Drawing on established frameworks and adapting them to the unique challenges of radiotherapy, this study outlines the steps taken to integrate TSEI effectively into clinical practice, ensuring both staff readiness and patient safety.

## Methods

Total skin electron irradiation was implemented in a German university hospital with a structured project plan oriented toward the Kotter eight-phase model [6] between the years 2022 and 2024. The Kotter model contains a sequential order of eight stages that characterize an organizational change process: *create a sense of urgency, building coalition, develop a vision, communicate the vision, enable action and remove barriers, generate short-term wins (pilot testing), consolidate, anchor change.*

The project core team involved two physicians, one technician, and one medical physicist. The stakeholder analysis regarding roles, interest, influence, involvement, expectations, and fears was performed oriented toward several examples from the literature and based on recommendations of, e.g., the German Federal Interior Ministry [7, 8], including an initial brainstorming within the core team and continuous development, e.g., by including concerns mentioned by the team.

Dosimetry was carried out on the basis of multiple recommendations, including AAPM Report No. 23 [3, 9]. Inhouse-constructed equipment necessary for treatment was included in the measurements to secure a sufficient dose distribution (e.g., an acrylic glass wall) and shielding of non-involved body parts (e.g., a lead helmet for the scalp). We accepted a maximum transmission of 10% of the applied dose to the scalp, nails, and eyelids, which was realized with a 1.2-mm layer of lead that was brought onto different materials. Pictures and information can be found in Supplementary Material C. To protect the scrotum in male patients, a commercially available testicle shield was used.

**Fig. 1:**
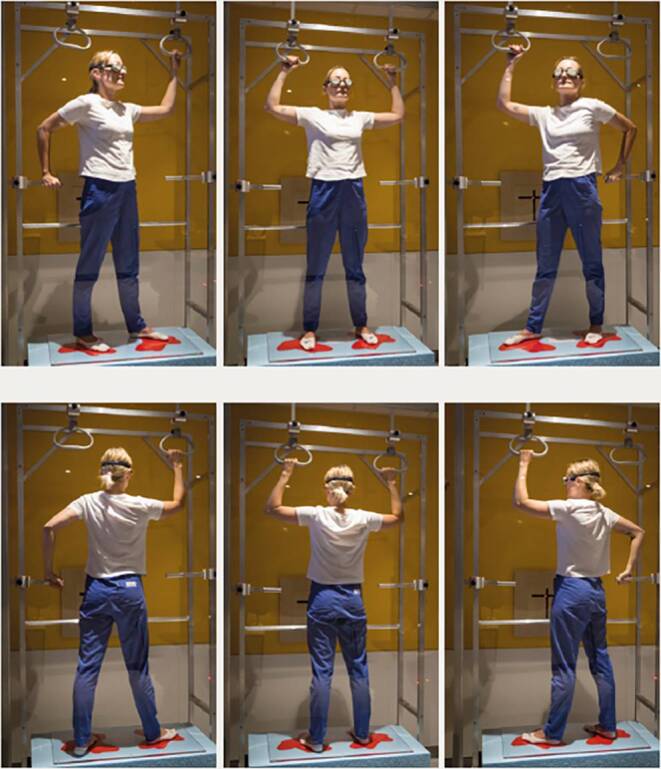
A person performing the six positions of the dual-fields technique during radiation

The TSEI was implemented following the six-dual-fields technique (Fig. 1; [9]). The protocol followed common protocols on low-dose radiation as published previously, with fractions of 2–4 Gy per day applied either to three (ventral or dorsal field every other day) or to all six fields per day, with maximum doses of 8–12 Gy with four treatments a week [10–12]. Areas of special risk due to high radiosensitivity are covered with lead if not affected by lymphoma. Additional fields are applied to regions of anticipated underdosage, such as the palms [13]. A prospective risk analysis for the method was conducted using a bowtie analysis [14]. As a detailed description of the protocol and results are beyond the focus of this article, reference is made here to the relevant publications [3, 9, 15, 16].

## Results

**Fig. 2:**
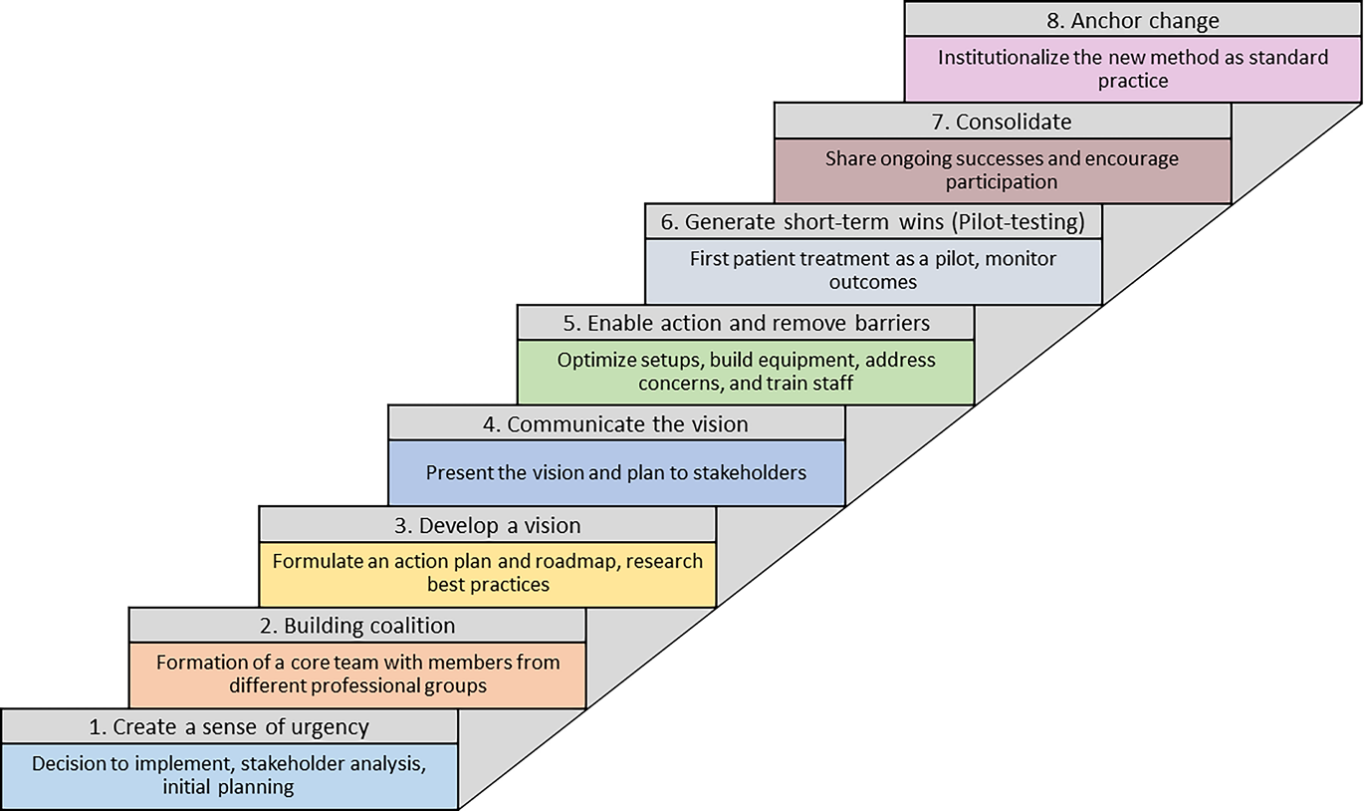
Flowchart of the eight phases of change as applied to total skin electron irradiation

The steps of implementation are shown in Fig. 2 and encompassed the following:

### Phase 1: create a sense of urgency

A special consultation hour for patients with cutaneous lymphoma was already established in the Department of Dermatology of the same hospital. For TSEI, those patients were sent to other departments in Germany, which was associated with long-distance travel for treatment and follow-up and impaired cooperation between radiation oncologists and dermatologists. At that time, only one of seven university hospitals in the wider region offered this special technique. In response to this, the departments of radiation oncology and dermatology jointly decided to aim for implementation of this technique.

A stakeholder analysis was initially conducted in this early phase to evaluate demands, concerns, and potential driving and restraining forces within the current context. It was continuously developed throughout the project. The table in Supplementary Material A offers an overview.

### Phase 2: building coalition

A core team was established to oversee the implementation process. This team included the chief medical officer of the department and a senior physician responsible for development of medical standards for TSEI as well as for coordination of education and contact with the Department of Dermatology. A medical physicist was responsible for dosimetric analyses and contributed to workflow development. A precision mechanic was responsible for the design and construction of the equipment for this method. A senior physician from the Department of Dermatology was nominated for collaboration.

### Phase 3: develop a vision

The team developed an action plan with tasks and subtexts under consideration of the stakeholders’ demands. Examples from the literature were reviewed concerning aspects of technical details, dosimetric concerns, and guidelines for patient treatment [3, 17, 18]. The process of implementation was subdivided into tasks, which allowed for allocation to different staff members and deadlines (Table 1).

**Table 1:** Categories and specific tasks for total skin electron irradiation implementation as conducted throughout the preparation an initial implementation during the first treatments

Category	Task
Dosimetry, quality assurance, and radiation protection	Organizing permissions for use of linear accelerators with cone-free electron radiation (authority)
	Dosimetric measurements with phantom
	Calculation/simulation and verification of the ideal setup of patient and linac gantry for aimed homogeneous dose distribution (treatment setup)
	Measurements with newly designed equipment for radiation protection of nails, eyelids, scalp, and scrotum
	Prospective risk analysis (repeat after 1 year)
Design, construction, and development of equipment	Design and construction of a frame for patient positioning
	Design and construction of radiation protection for nails, eyelids, and scalp
	Purchase of a commercially available testicle shield
	Marking the radiation room to ensure accurate reassembly of the radiation frame
	Assessment of equipment by internal experts for safety at work and hospital hygiene
	First test of equipment with team of RTTs; feedback and enhancement of design and construction
Development of clinical standards and documentation	Definition of a clinical standard operating procedure according to guidelines and literature
	Design of a form for treatment prescription
	Design of a form for daily treatment delivery documentation
	Provide German version of mSWAT questionnaire for patient assessment
	Provide German versions of questionnaires on quality of life for patient assessment and treatment outcome
	Standard for patient transfer between departments of dermatology and radiation oncology
Education	Introduction of concept and roadmap for the whole team (presentation)
	Training the ward team with a focus on patient monitoring and skin care
	Education session for RTTs with a focus on patient setup
	Education session for physicians with a focus on treatment parameters, patient assessment, and surveillance
	Education session for staff of the dermatology department with focus on treatment techniques and technical and clinical considerations for patient selection
Communication	Design of a flyer for patient information
	Update of information on the hospital website
	Setting up a cooperation with the hospitals’ department for public relation
Feedback	Presentation of clinical cases in staff meetings
	Including one-to-one feedback in employee appraisal
	Demanding feedback from the Department of Dermatology through personnel communication and follow-up reports

*RTT* radiation therapist; *mSWAT* modified severity assessment tool

### Phase 4: communicate the vision

The complete departmental team was introduced to the background and vision for implementing the method through a presentation at a quarterly departmental meeting. The clinical background and purpose were emphasized, and the timeline was presented. The team of physicians had a further presentation about the pathology of cutaneous lymphoma, current practices, and study results.

### Phase 5: enable action and remove barriers

The dosimetric analyses allowing for TSEI setup in the respective department and the respective licensing was performed alongside phases 3–4. Simultaneously, an initial design for the patient setup frame was constructed. To enable participation of different staff members, several on-site meetings in the treatment room with the physician and radiation therapy technologist (RTT) team together with the core team were held. The patient setup with the in-house frame for positioning as well as the handling of other equipment was discussed to gather feedback concerning safety and usability for patients and staff and optimization of the whole process. The discussion and the emerging fears of possible sources of error were incorporated into the prospective risk analysis (Supplementary Material B). An educational session for the ward nurses was held concerning patient surveillance and a new skin care plan. Another educational session was performed for the medical staff of the Department of Dermatology. Within this educational session, a process standard for communication within the two departments was discussed and evolved. Written standard procedures were saved in the local data store and were available for every team member.

### Phase 6: generate short-term wins (pilot testing)

The first patient (male, 67 years, with Sézary syndrome) received his TSEI treatment in January 2023. A kickoff meeting between RTTs and the core project team was held right before treatment to recognize and rectify possible ambiguities or errors. Clinical parameters (e.g., blood cell count, electrolytes, glomerular filtration rate, blood pressure, temperature) were monitored during the treatment course. The scores for quality of life were recorded and photographs of the skin appearance were taken. Treatment effects were communicated to all involved team members.

### Phase 7: consolidate

The successful treatment of the first and subsequent patients was communicated in a departmental meeting to inform all team members about the new structures and results. A nearly constant flow of patient appointments was generated through close interaction with the Department of Dermatology. Information about the treatment was compiled into a flyer (Supplementary Material D), informative website texts, and a journal article, all designed to be understandable for laypersons [19]. We experienced very good protocol adherence by RTTs and physicians, with close feedback loops ensuring a constantly high quality of care.

### Phase 8: anchor change

After the first treatment courses with a fixed team, more and more team members were integrated to learn the setup, workflow, and clinical surveillance. Reports about successfully completed treatments as proven by feedback from patients and referring dermatologists were communicated during internal meetings, congresses, and specific conferences. Based on ongoing studies and results, we included new dose concepts or therapeutic sequences, such as combinations of TSEI and brentuximab [20, 21]. The prospective risk analysis was continuously reviewed.

## Discussion

Total skin electron irradiation is a powerful treatment option that offers an improvement in quality of life and reduces the overall disease burden for patients with cutaneous lymphoma [10, 22]. Because the introduction of this technique is challenging in terms of education, creating structures, and equipment, it is not commonly available in most radiation oncology departments [23]. We often face similar challenges when introducing new technologies and treatments in radiotherapy, whereby the introduction of TSEI is particularly suitable as an example due to the involvement of many professional groups and process aspects. If an institution decides to introduce similar challenging innovations, it should adhere to a structured management plan for reasons of cost-effectivity and sustainable implementation [24, 25].

A stakeholder analysis provides a solid basis for the players in a change process and has been described in terms of its importance for change management [26]. Stakeholders with a strong interest, involvement, and influence must be sufficiently engaged, as they have the potential to make or break the entire project. In our case, the highest level of involvement and influence was attributed to RTTs and physicians. Therefore, the core project team ensured their strong participation throughout the entire project. Participation for all team members ranged from pure information to co-decision-making. According to the so-called participation efficiency hypothesis, fears among staff are replaced by a feeling of control, and change is not seen as a restriction of freedom but rather as a self-determined opportunity to shape the process. This can lead to employees contributing their specialized expert knowledge to the change process, thereby improving the quality of decision-making [27]. Furthermore, a high level of involvement allows for in-detail discussions of the process from different perspectives, quality and safety improvements, and a comprehensive risk analysis [28]. With ongoing innovations in radiation oncology, we expect an ongoing need for change management within multiprofessional teams, e.g., for implementation of artificial intelligence-based solutions [29].

## Conclusion

By leveraging established change management frameworks and adapting them to the specific context of radiotherapy, the clinic successfully integrated TSEI, thus enhancing its treatment capabilities and improving patient outcomes.

## Supplementary Information

Supplementary Material A Stakeholder analysis

Supplementary Material Table B Risk analysis

Supplementary Material C Photos of equipment

Supplementary Material D Flyer with information for patients
