# Supplementary material for: Implementing total skin electron irradiation in radiotherapy: a structured change management approach
Source: Strahlenther Onkol. 2025 May 14;202(1):68–73. doi: 10.1007/s00066-025-02408-w (PMC12819445; doi:10.1007/s00066-025-02408-w)
Supplement: Supplementary file 1 — Supplementary Material A Stakeholder analysis [file 66_2025_2408_MOESM1_ESM.pdf]

Table A: Stakeholder roles, involvement, interest, influence, and concerns in TSEI Implementation. RTTs = radiation therapists

Stakeholder	Role and level of involvement	Level of influence	Level of interest	Expectations and demands	Fears
Internal					
Patients	Receiving treatment; intermediate level of involvement	low	high	Safe delivery of care, adequate timeframes for care (including waiting times for appointments), care delivery close to their hometown, safe interaction between treating parties	Unsafe care, insecurity concerning treatment pathways, side effects or unsafe surveillance of side effects
Radiation oncologists	Indication setting, patient selection, treatment planning and surveillance of treatment and side effects; high level of involvement	high	high	Opportunity to deliver adequate care, Opportunity to learn new treatment techniques, adequate training for technique and patient care	Overstrain with new technique, schedule collision between "regular" and TSEI-patients, overstrain with side effects of TSEI, unsafe care through lapses or errors in treatment planning
RTTs	Radiation delivery, technically correct implementation and patient setup; high level of involvement	high	high	Opportunity to deliver adequate care, Opportunity to learn new treatment techniques, adequate training for technique, opportunity to work with a technique which is not commonly available in other departments	Overstrain with new technique, burden of organizing schedule of TSEI patients, burden of installing and demount of equipment
Ward staff/nurses	Patient surveillance during RT; intermediate level of involvement	intermediate	intermediate	Opportunity to learn about new pathology, training for adequate skin care for special implication	Overstrain with side effects of new treatment technique
Medical Physicists and engineers	Dosimetric preparation of RT technique, calculations,	high	high	Accuracy of dosimetry and maximization of the	Over- or underdosing of individual body parts due to incorrect positioning of the patient

	measurements for dosimetry high level of involvement			homogeneity of the dose distribution	
Department management	Coordination of the project, identification of costs for materials, education, public relation and RT treatments vs. compensation; intermediate level of involvement	high	high	Opportunity to offer treatment, which is not commonly available (unique selling point), improvement of cooperation with department of dermatology, keeping up a good team spirit/motivated team members, creating a safe process for team members and patients	Direct costs through investment in licenses and equipment, indirect costs through “schedule collision” between TSEI treatments and other treatment courses, indirect costs through releasing the project team from “usual” tasks in daily practice for implementation of the new technique, negative interference among staff, unsafe conditions leading to errors during RT delivery and patient harm, unsafe surveillance or therapy of side effects
External					
Dermatologists	Identification of patients who can benefit from TSEI treatment, contact RT department for jointly treatment of patients and joint follow up; intermediate level of involvement	low	high	Timely scheduled treatments, safe delivery of treatment, close interaction and jointly surveillance of patients, available information about RT processes and structures	Unsafe treatment, unsafe surveillance of side effects for jointly patients, inadequate timeframes/waiting times for patients, not clearly defined roles/responsibilities/procedures to interact with RT department
Other radiation therapy departments	Identification of patients who can benefit from TSEI treatment, contact RT department for patient transfer; low level of involvement	low	intermediate	Timely scheduled treatments, safe delivery of treatment, available information about processes and structures of TSEI in the department	inadequate timeframes/waiting times for patients, not clearly defined roles/responsibilities/procedures to interact with RT department
University Hospital/Organization	Offering structures and logistics for interdisciplinary cancer care; low level of involvement	intermediate	intermediate	Opportunity to offer treatment, which is not commonly available (Unique selling point), improved reputation of hospital, safe	Failure of implementation leading to bad reputation, unsafe conditions leading to errors during treatments

				processes for team and patients	
Public	Receiving information about TSEI and cutaneous lymphoma; low level of involvement	low	intermediate	Receiving up to date-information about current practices, how they are implemented and applied, receiving information about innovations in public hospitals	none
Radiation protection authority	Providing the license for the use of the linear accelerator for TSEI; low level of involvement	high	low		none
