# Supplementary material for: Implementing total skin electron irradiation in radiotherapy: a structured change management approach
Source: Strahlenther Onkol. 2025 May 14;202(1):68–73. doi: 10.1007/s00066-025-02408-w (PMC12819445; doi:10.1007/s00066-025-02408-w)
Supplement: Supplementary file 2 — Supplementary Material Table B Risk analysis [file 66_2025_2408_MOESM2_ESM.pdf]

Prospective risk analysis for TSEI - Part of the publication Change management in Radiotherapy - A real-world example of implementing a new method by A. Baehr et al.

Event	Causes	Preventive measures	Discovery mechanisms	Consequences	Measures after the event has reached the patient
accessories for shielding not used	accessories cannot be found	standardised storage space for accessories	review of the photo documentation	overdosage of areas to be covered	calculation of the real dosage, equalisation by adjusting the radiation dose
	indication for the use of accessories not known to the physician doctor who prescribed the treatment	training of doctors on the use of accessories	review of the instructed accessories by second physician		
	accessories not entered in the order	comparison of accessories with clarification documentation and photo by second physician	review of the instructed accessories by second physician	overdosage of areas to be covered	calculation of the real dosage, equalisation by adjusting the radiation dose
gloves with lead pads do not sufficiently cover the nails	gloves selected too large	gloves available in three sizes	feedback by patient or view of photo documentation	increased dose to nails	if not all fractions have been applied, additional fractions with a different glove size
	gloves not put on correctly	donning with visual inspection by staff	review of the photo documentation		if not all fractions are given, further fractions with correct handling
safety goggles do not adequately cover the eye area	goggles not adequately adjusted	size-adjustable strap for adjustment	review of the photo documentation	increased dose on eyelid, eye and gl. lacrimalis	if not all fractions are given, further fractions with correct handling
	goggles not fitted correctly	Donning with visual inspection by staff			
lead helmet does not adequately cover the hair follicles	helmet too big	adjustment by using surgical bonnets under the helmet	Feedback from patient or view of photo documentation	increased dose on hair follicles	if not all fractions are given, further fractions with correct handling
	helmet too small	helmet designed very large	photo documentation/clinical impression		
incorrect specifications of the plan parameters (gantry, apertures, MU)	overlooked	physicists` peer review	review second physician or physicist	ounder/overdosage	measurement/calculation of the actual dosage, if necessary equalisation by adjusting the following fractions or the enddose

deviating specifications of the HDTSE beam type (plan parameters)	overlooked	physicists` peer review	no beam release possible	none	
machine-related dosimetric deviation	machine-related	dose measurement	14-day dosimetry check as part of the weekly check-up	under/overdosage max+/- 2%	measurement/calculation of the actual dosage, if necessary equalisation by adjusting the following fractions or the enddose
			daily MPC + quarterly measurement		
machine-related geometric deviation of apertures	machine-related	dose measurement	daily MPC + half-yearly measurement	minor under/overdosage	
patient injured during access to the radiation rack	immobile/geriatric patient	screening of patients with information regarding mobility		accident, fall, injury	physical examination and indicated care
	due to nervousness or accidentally	patients are accompanied by staff to their final position			
Incorrect positioning of the radiation rack (SSD/plate distance)	overlooked	markings for and on the trolley	adjustment with specification	minor under/overdosage	measurement/calculation of the actual dosage, if necessary compensation by adjusting the following fractions or the enddose
incorrect vertical patient position	incorrect information on patient size, specification overlooked	comparison with specification by RTTs	comparison with specification by RTTs	minor under/overdosage	
field (A-M) is omitted	forgotten/overlooked	standardised sequence of the individual fields during irradiation	oeer Review by two RTTs	underdosage	measurement/calculation of the actual dosage, if necessary equalisation by adjusting the following fractions or the enddose
	restless environment, lack of concentration	individual documentation of the fields			
		minimisation of the number of people in the control room			
field (A-M) is omitted	no change in patient position after radiation of the field	documentation of the fields individually	peer Review by two RTTs	overdosage	measurement/calculation of the actual dosage, if necessary compensation by adjusting the following fractions or the enddose
	restless environment, lack of concentration	minimisation of the number of people in the control room			
		telephone is answered by 2nd person			

patient cancels irradiation	fatigue	Screening of patients during information regarding their ability to stand for 30 minutes		underdosage	addition of missing fields
	feeling cold	patient wears long but loose cotton clothing			
	urge to urinate	patient is instructed to go to the toilet beforehand during explanation			
local tumour irradiation does not take place	local radiotherapy refused by patient	Informing the patient about the need for local tumour irradiation	first RT accompanied by senior physician	underdosage	subsequent radiation
	local radiotherapy not prescribed/forgot to prescribe	review of the prescription (cover sheet and initial consultation) by second physician			
	indication for local tumour irradiation wrongly not given	training of doctors on the indication for local tumour irradiation			
saturation of the covered skin areas does not take place	indication for saturation of the covered skin areas incorrectly not given	Review of the prescription (cover sheet and initial consultation) by a second physician	first RT accompanied by senior physician	underdosage	subsequent radiation
	aaturation not prescribed/forgotten to prescribe	training of doctors on the indication of saturation			
pre-irradiation of the skin is not observed	no query during the first patient visitation	compliance with existing SOPs	review of the documentation by second physician	overdosage	adjustment of the enddose
underdosage of facial areas	incorrect head position during 60° fields	special note during education (head position analogue to foot position)	peer review by two RTTs	underdosage	subsequent radiation
		advice to patient during positioning			

underdosage of hand areas	incorrect hand position on handholds	special note during education	peer review by two RTTs	underdosage	subsequent radiation
		advice to patient during positioning			
underdosage of the axilla	arm position less than 90°	special note during education	peer review by two RTTs	underdosage	subsequent radiation
		adequate setting on the radiotherapy frame			
insufficient skin prophylaxis	lack of knowledge about prophylaxis	SOP contains information on skin care	supervision by senior physician	skin reactions	adaptation of skin care
skin toxicity not adequately treated	patient not adequately supervised	training of inpatient physicians	supervision by senior physician	infections, dehydration	therapy according to guidelines
	lack of knowledge about skin therapy	further training, consultative care by dermatology			dermatology consultation, therapy according to guidelines
tumour lysis syndrome not diagnosed	failure to take blood samples	SOP includes information on continous surveillance for hematologic parameters	supervision by senior physician	renal dysfunction	use of rasburicase, hydration
