# Supplementary material for: Implementing total skin electron irradiation in radiotherapy: a structured change management approach
Source: Strahlenther Onkol. 2025 May 14;202(1):68–73. doi: 10.1007/s00066-025-02408-w (PMC12819445; doi:10.1007/s00066-025-02408-w)

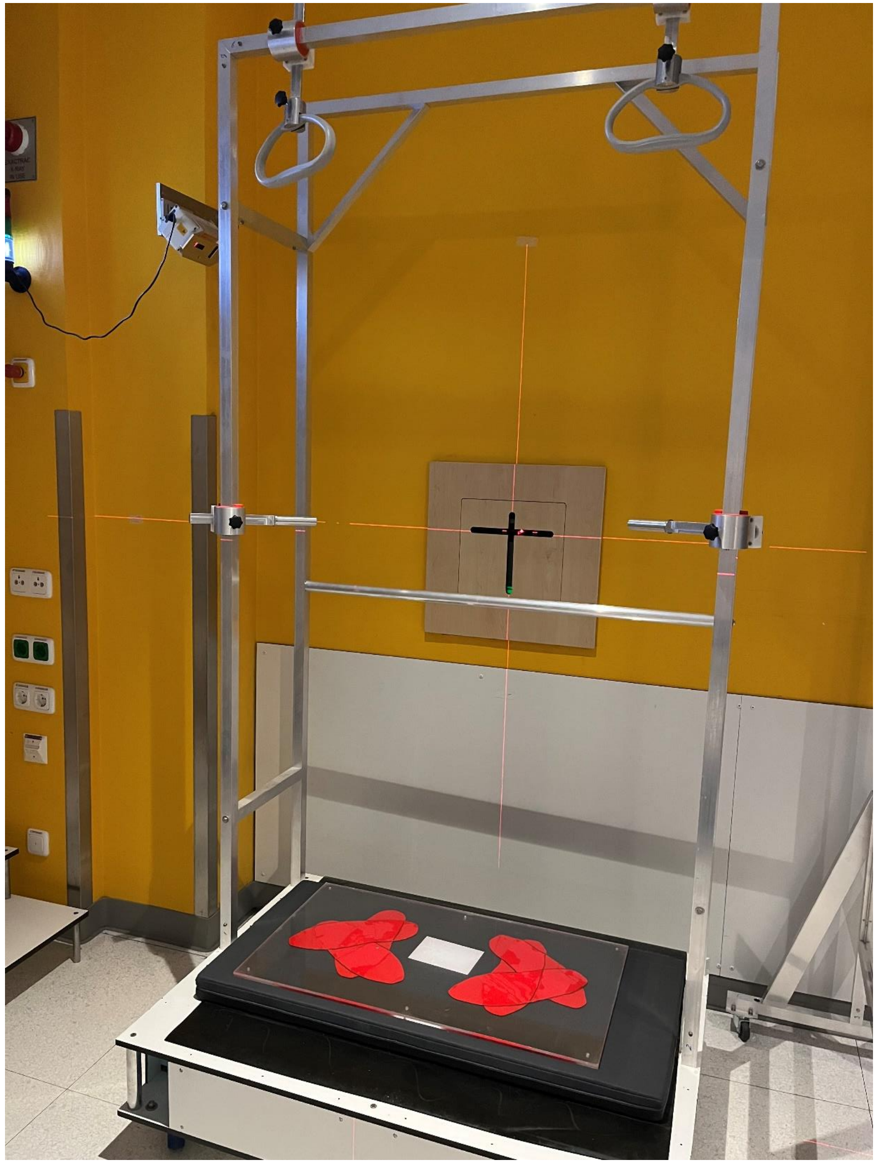

Platform and frame for carrying out the irradiation:

The patient performs six different positions during treatment which is facilitated foot-shaped red markings on the floor, two side hand grips and two top handles. All handles can be adjusted in height and rotation. The height of the floor can be varied according to the patient's height using polystyrene blocks of 5 cm each.

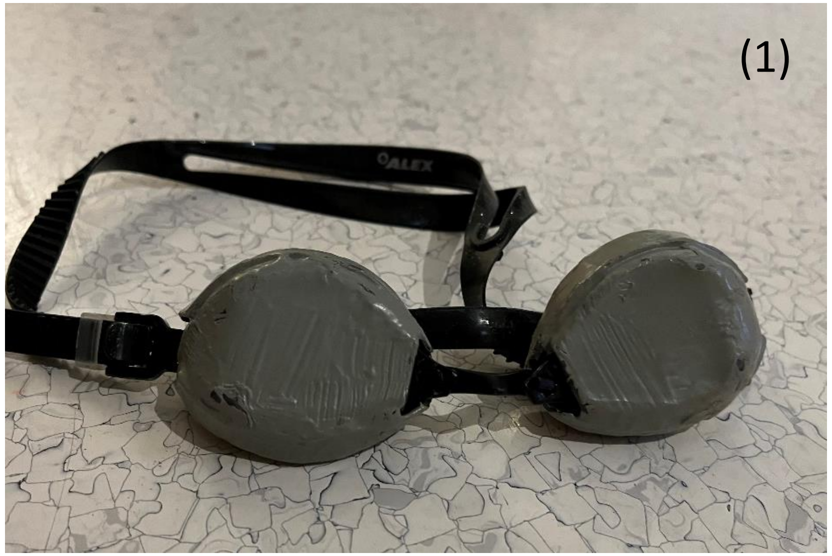

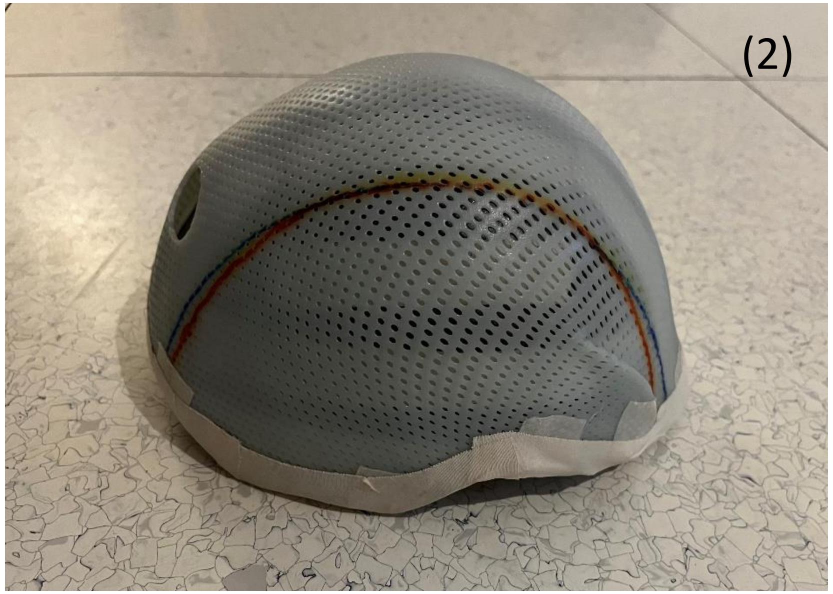

Equipment for shielding:

Googles (1) and helmet (2) each with a 1.2 mm thick layer on lead brought to sports swimming goggles as commercially available (1) and a helmet molded for this purpose from thermoplastic material as used in radiotherapy for mask preparation (2).

Part of the publication Change management in Radiotherapy - A real-world example of implementing a new method by A. Baehr et al.

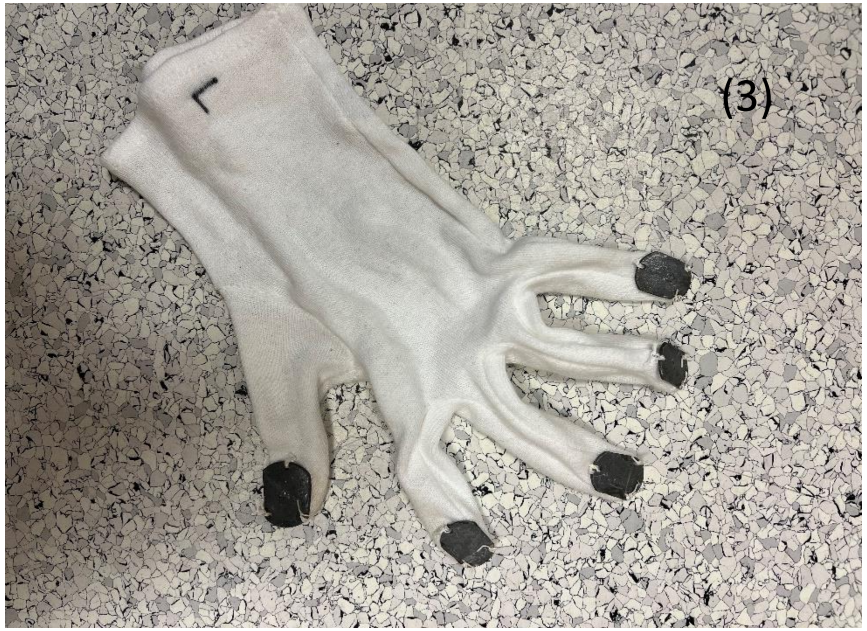

Equipment for shielding:

Gloves (3) and shields for toe nails (4) each with a 1.2 mm thick layer on lead brought to cotton gloves (3) and and modulated as caps that can be placed over the toes (4).

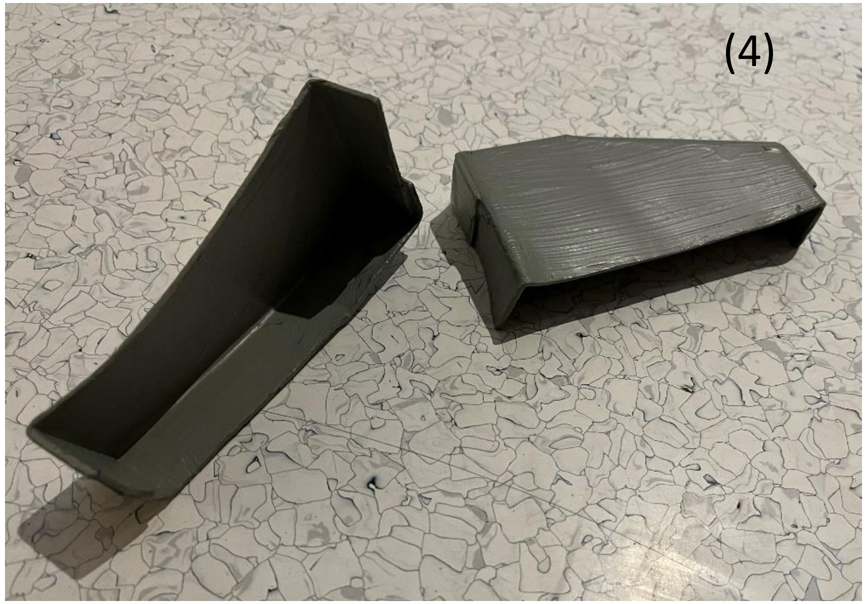

Supplement: Supplementary file 3 — Supplementary Material C Photos of equipment [file 66_2025_2408_MOESM3_ESM.pdf]
