# Supplementary material for: Implementing total skin electron irradiation in radiotherapy: a structured change management approach
Source: Strahlenther Onkol. 2025 May 14;202(1):68–73. doi: 10.1007/s00066-025-02408-w (PMC12819445; doi:10.1007/s00066-025-02408-w)
Supplement: Supplementary file 4 — Supplementary Material D Flyer with information for patients [file 66_2025_2408_MOESM4_ESM.pdf]

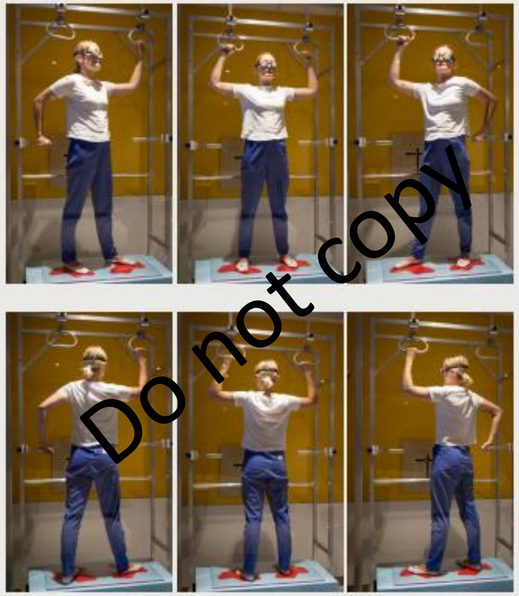

Do not copy

Die Ganzhautbestrahlung wird in sechs Positionen im Stehen durchgeführt. Dabei können je nach Ausprägung der Krankheit täglich alle oder täglich die Hälfte aller Positionen bestrahlt werden. Einige Körperteile können in der stehenden Position nicht ausreichend mit Strahlendosis versorgt werden (z. B. Fußsohlen). Diese werden zusätzlich mit einer sogenannten Aufsättigungsbestrahlung behandelt.

Do not copy

Unsere Kontaktdaten

Ihre Ansprechpartnerinnen

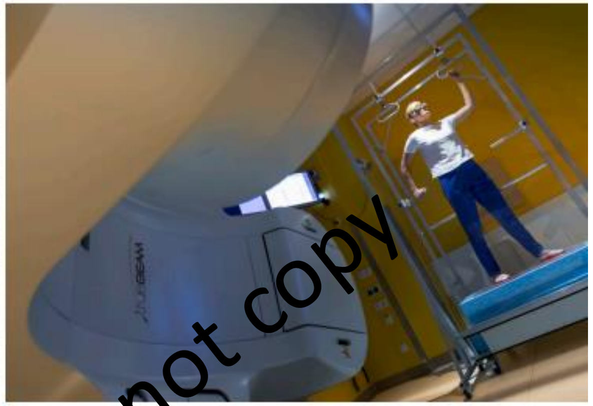

Do not copy

Für Patient:innen

Ganzhautbestrahlung
bei kutanen Lymphomen

**Flyer with information for patients:**

This flyer in German language contains information for patients concerning background, radiation technique, process and possible side effects.

## Liebe Patient:innen,

pro 100.000 Menschen erkrankt eine Person jährlich in Deutschland an einem Kutanen Lymphom, einer seltenen Form des Hautkrebses, bei der sich bestimmte Immunzellen unkontrolliert in der Haut vermehren. Besonders häufig kommen die Lymphome "Mycosis fungoides" und das "Sézary-Syndrom" vor.

Mittels individuell konzipierter Therapie, zu der auch die Ganzhautbestrahlung gehört, möchten wir Betroffenen zu neuer Lebensqualität verhelfen.

Mit diesem Flyer erläutern wir Ihnen den Ablauf einer Ganzhautbestrahlung. Und wir möchten Sie ermutigen, denn: Strahlentherapie erweist sich als besonders wirksam.

Wir wünschen Ihnen eine gute Genesung!

Do not copy

### Bestrahlung ist ein Bestandteil der Therapie

Jede Therapieempfehlung sprechen wir für die:den jeweilige:n Patient:in individuell und im interdisziplinären Team aus.
Die Bestrahlung einzelner Hautläsionen kann zeitgleich zur medikamentösen Behandlung erfolgen.
Bei Patient:innen, die unter laufender medikamentöser Therapie nicht oder nicht ausreichend ansprechen, ist die Bestrahlung besonders wirksam.

### Ablauf der Ganzhautbestrahlung

Die Bestrahlung der gesamten Hautoberfläche erfolgt mit Elektronen. Weil diese nur wenige Zentimeter in die Haut eindringen, wird die Strahlenwirkung auf die Hautoberfläche beschränkt. Die Therapie erfolgt stationär in unserer Klinik über einen Zeitraum von zwei bis drei Wochen. So garantieren wir eine ausreichende Überwachung und Unterstützung bei der Hautpflege.

Do not copy

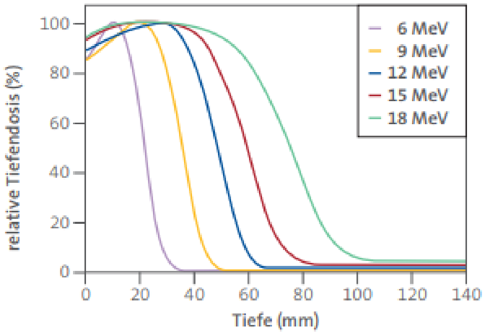


Darstellung der wirksamen Dosis (in %) von Eektronenstrahlung auf Hautlymphome. Die Wirksamkeit nimmt mit der Gewebetiefe (in mm) ab. Bei höheren Energien erreicht man in tiefen Geweben eine bessere Wirksamkeit.

Die Nachsorge führt das Team der Klinik für Dermatologie gemeinsam mit dem Ambulanzzentrum des UKE durch.

### Ziel der Ganzhautbestrahlung

- Rückgang von Hautrötung, Schuppung, Entzündungen und Juckreiz im Rahmen der Erkrankung
- Zunahme von Lebensqualität
- Verbesserung der Blutparameter

### Gute Verträglichkeit

Die Bestrahlung der Haut wird in der Regel sehr gut vertragen. Häufige Nebenwirkung sind vorübergehende Schuppung und Rötung der Haut sowie Juckreiz. Darüber hinaus kann es zu einer Veränderung bestimmter Parameter des Blutbildes und einem Anstieg der Nierenretentionsparameter kommen. Da sich die Hautbarriere erneuert, ist auf eine ausreichende Flüssigkeitsaufnahme zu achten.

Do not copy

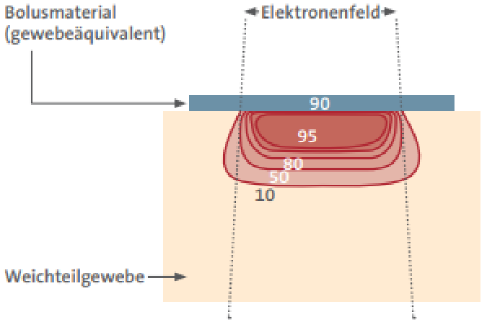


Das Elektronenfeld trifft zunächst auf ein Bolusmaterial, dann auf die Hautschichten. Dadurch kann an der oberen Hautschicht eine sehr wirksame Dosis (in % Wirksamkeit) erreicht werden.

## Flyer with information for patients:

This flyer in German language contains information for patients concerning background, radiation technique, process and possible side effects.
